# Evaluation of the Efficacy of Three Antagonistic Bacteria Strains in the Management of Fire Blight

**DOI:** 10.3390/ijms26094438

**Published:** 2025-05-07

**Authors:** Jianhui Zhang, Zhidong Zhang, Yue Wen, Jing Zhu, Abudusufuer Wufuerjiang, Jia Tian

**Affiliations:** 1 College of Horticulture, Xinjiang Agricultural University, Urumqi 830052, China; 18730698113@163.com (J.Z.); wenyue900701@163.com (Y.W.); sufuer715@163.com (A.W.); 2 Xinjiang Key Laboratory of Special Environmental Microbioogy, Xinjiang Academy of Agricultural Sciences, Urumqi 830091, China; zhangzheedong@sohu.com (Z.Z.); zhujing2020@hotmail.com (J.Z.)

**Keywords:** fire blight, Korla Xiangli, antagonistic bacteria, enzyme activity, control efficacy

## Abstract

Fire blight, caused by *Erwinia amylovora*, poses a significant threat to the sustainable development of the Korla Xiangli (*Pyrus×sinkiangensis*. Yu) industry. In this study, we used multiple experimental approaches to comprehensively evaluate the efficacy of three antagonistic bacterial strains—namely, Mg-7 (*Leuconostoc mesenteroides*), Rt-10 (*Alcaligenes faecalis*), and Rt-11 (*Bacillus siamensis*)—in controlling fire blight. In vitro plate inhibition assays revealed that Mg-7 exhibited the largest inhibition zone diameter, exceeding Rt-10 and Rt-11 in this respect, suggesting its strong antifungal potential. In therapeutic tests conducted on detached leaves, Mg-7 achieved the highest control efficiency, 60.39%, while Rt-10 demonstrated the greatest efficiency (76.96%) in protective tests. Conversely, in therapeutic trials focusing on detached branches, Mg-7 showed a control efficiency of 45.90%, whereas Rt-11 exhibited the highest efficiency, 86.27%, in protective trials. Furthermore, in vitro evaluations indicated that the Mg-7 treatment significantly reduced the lesion spread area. Enzymatic analyses revealed that, in the leaf protection assay, catalase activity (CAT) demonstrated significant increases of 65.56%, 85.46%, and 45.55% under the Mg-7, RT-10, and RT-11 treatments, respectively, when compared with the EA control group on day four. Correspondingly, in the branch protection assay, polyphenol oxidase (PPO) activity displayed marked elevations of 62.84%, 52.06%, and 82.69% under identical experimental conditions at the same time point. These treatments not only upregulated antioxidant enzyme activities but also significantly reduced malondialdehyde (MDA) content, effectively mitigating oxidative damage while enhancing foliar and branch resistance to fire blight infection. Field trials conducted in outdoor orchards confirmed that the Mg-7 bacterial suspension provided more effective and stable control against fire blight than Rt-10 and Rt-11. Overall, Mg-7 shows significant potential for use as a biocontrol agent for managing fire blight because of its high efficacy, stability, and ability to enhance plant defense responses.

## 1. Introduction

Fire blight, a serious disease caused by the bacterium *Erwinia amylovora* [1], has been a longstanding concern for rosaceous fruit trees since its identification in North America in 1780 [2]. This disease spreads through wind, rain, and insect activity, infecting leaves, flowers, and fruits. The systemic nature of fire blight can lead to rapid and severe damage, often resulting in the death of the affected plants, presenting significant challenges for its effective control and eradication [3].

The Korla Xiangli (*Pyrus × sinkiangensis*. Yu), a well-regarded fruit tree in the Rosaceae family, is celebrated for its thin skin, fine flesh, sweetness, and juiciness, making it a valuable agricultural product in Xinjiang [4]. Unfortunately, fire blight outbreaks have become increasingly prevalent in the Ili Kazakh and Mongolian Autonomous Prefectures of Xinjiang since 2016 [5]. By 2017, approximately 6700 hectares were affected, leading to a substantial 30–50% decrease in the production of Korla Xiangli and the destruction of nearly 1 million pear trees [6]. Consequently, the impact on the planting area has been severe, with a reported reduction of 4100 hectares by 2020, underscoring the economic ramifications of fire blight within this region [7]. At present, fire blight affecting Korla Xiangli production is still mainly controlled using chemical pesticides (such as streptomycin, oxytetracycline, kasugamycin, etc.), combined with agronomic measures such as water and fertilizer management and enhancing tree vigor to improve the trees’ disease resistance. However, the control effect is limited. Moreover, long-term reliance on chemical pesticides has allowed many fire blight pathogens to develop resistance to commonly used pesticides; moreover, this method is not environmentally friendly, posing safety risks to humans and livestock [8]. In light of these challenges, the exploration of antagonistic bacteria and their active substances to develop biological agents for disease control offers multiple advantages. Such alternatives are characterized by strong selectivity, a low likelihood of pathogen resistance, safety, efficiency, and environmental friendliness [9]. Therefore, exploring the use of beneficial micro-organisms with inhibitory effects on pathogens has become a prominent trend in the green development of modern agriculture and pesticide research and development. Finding efficient antagonistic biological agents for fire blight is a key factor for controlling it.

In recent years, there have been notable advancements in the research on and practical application of biological control methods for fire blight. Zeller et al. [10] identified the *Erwinia herbicola* strain 89 from apple flowers and leaves. It demonstrated a control efficacy of nearly 70% against fire blight. Vanneste et al. [11] conducted a comparative trial on flowering plants by using various biological agents and found that *Erwinia herbicola* strain Eh252 exhibited a control effect comparable to that of streptomycin. Additionally, Sharifazizi et al. [12] isolated *Pantoea* sp. (Pa21) and *Pseudomonas fluorescens* (Ps170) from pear trees and found that both strains displayed significant efficacy in biological control experiments. Notably, Pa21 achieved a control efficiency of up to 83% with respect to immature fruits, while Ps170 recorded a control efficiency of 92% with respect to detached flowers. Moreover, the plant lactic acid bacterium PC40, isolated from pear and apple flowers, exhibited substantial colonization capacity and maintained a stable antibacterial capacity under high-humidity conditions for up to a week [13]. The enzymatic defense mechanisms of plants also play a crucial role in their resistance to pathogen infection, involving key defense enzymes such as superoxide dismutase (SOD), peroxidase (POD), phenylalanine ammonia-lyase (PAL), and CAT, which significantly influence disease resistance [14]. Recent studies have provided further insight, as *Alcaligenes faecalis* has been found to secrete a hydroxylamine compound with significant inhibitory effects on plant pathogens [15]. Liu et al. [16] screened *Leuconostoc mesenteroides* strain AP7 from fermented vegetables and found that it exhibited excellent antibacterial performance against foodborne pathogens, with particularly notable inhibitory effects. In research on the control of peanut crown rot, *Bacillus siamensis* strain ZHX-10, applied through root irrigation, was observed to promote plant growth and effectively inhibit the crown rot pathogen, with a control efficiency of up to 50.04% [17].

In this study, we investigated the biocontrol potential of three antagonistic bacteria—namely, Mg-7, Rt-10, and Rt-11—which were previously isolated by our research group and are known for their antagonistic effects on fire blight. The antibacterial properties of these strains were validated through plate confrontation experiments. For the indoor protective and therapeutic trials, detached branches and leaves of the Korla Xiangli were utilized. The efficacy of the treatments was assessed by measuring the incidence of disease and monitoring changes in enzyme activity. Additionally, outdoor evaluations were performed by applying bacterial suspensions in an orchard highly susceptible to fire blight to determine their control efficacy under field conditions. This research improves our understanding of the application of the three antagonistic strains Mg-7, Rt-10, and Rt-11 in the prevention and control of fire blight in Korla Xiangli, surpassing the traditional single experimental mode and successfully translating achievements from the laboratory to the field through a “from in vitro plate confrontation to in vitro organ protection/treatment trials and finally to field trial verification” model. Moreover, based on information collected from the dynamic monitoring of enzyme activities, the biological control mechanisms of the three antagonistic bacteria with respect to plant antioxidant enzymes were revealed. This study offers valuable resources for managing this disease and supporting the sustainable and high-quality development of the Korla Xiangli industry.

## 2. Results

### 2.1. Molecular-Biology-Based Verification Results for Fire Blight Disease

**Figure 1:**
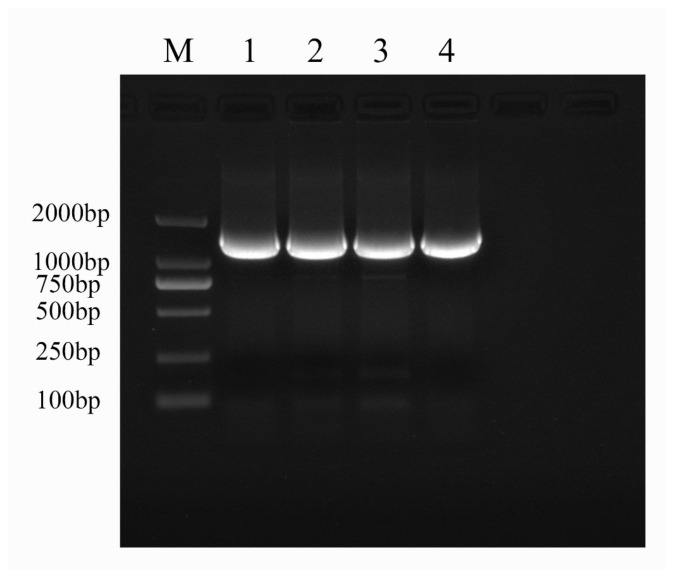
PCR amplification results regarding *Erwinia amylovora* Xea.

**Figure 2:**
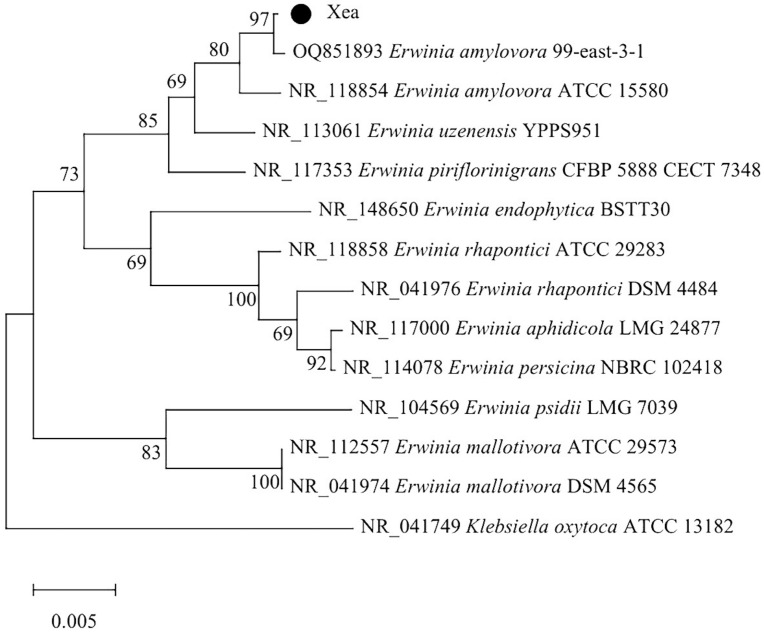
The phylogenetic tree of Xea based on a 16S rRNA sequence.

PCR amplification was conducted on the Xea strain, utilizing universal primers 27F and 1492R, resulting in a target product of approximately 1500 bp. This size aligns with the anticipated fragment length (Figure 1). Subsequent analysis of the sequencing data were performed through NCBI Blast, leading to the identification of the strain as *Erwinia amylovora* strain ATCC 15580. To further validate these findings, similar sequences were aligned using DNAStar, and the most closely related model strain genes were selected for comparison. A phylogenetic tree was subsequently constructed, employing the neighbor-joining method in MEGA 7.0 (Figure 2).

### 2.2. Morphological Characteristics of Erwinia amylovora

**Figure 3:**
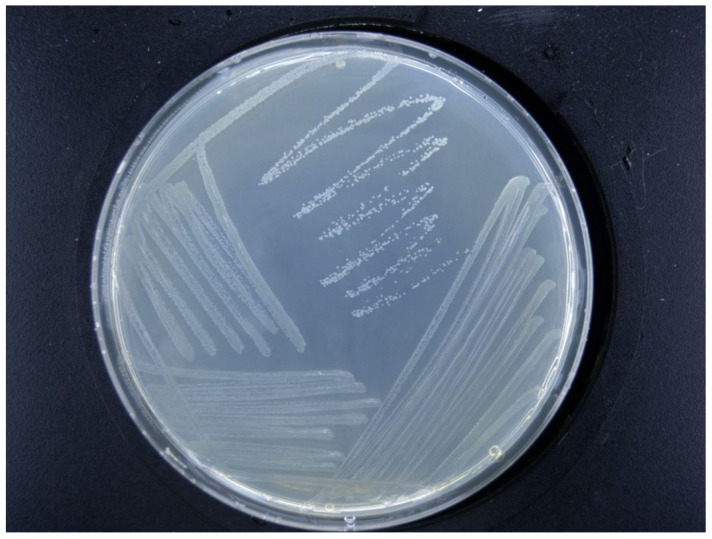
Morphological characteristics of *Erwinia amylovora* (Xea) on the NA medium.

The colonies were grown in an NA medium at 28 °C for 24 to 48 h. The diameter of the colony was approximately 1 to 3 mm, and it was round or nearly round, with neat edges and obvious protrusions (a convex surface). It was initially milky white and semi-transparent. As the culturing time extended, it gradually turned light yellow or pale amber. Some strains showed a slightly yellowish cream color. Moist and smooth, with luster and no wrinkles on the surface, the colony had relatively high viscosity and was opaque. No fluorescent pigment was produced, and there was no hemolysis, features in line with the morphological characteristics of *Erwinia amylovora* (Figure 3).

### 2.3. Pathogenicity of Xea Strains Causing Fire Blight

**Figure 4:**
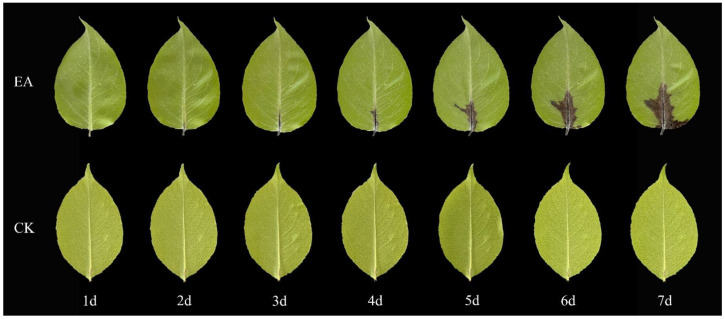
The susceptibility of Korla Xiangli leaves one to seven days after inoculation with fire blight. Note: EA represents the treatment consisting of a single inoculation with *Erwinia amylovora*, and CK represents the treatment consisting of a single inoculation with clear water.

**Figure 5:**
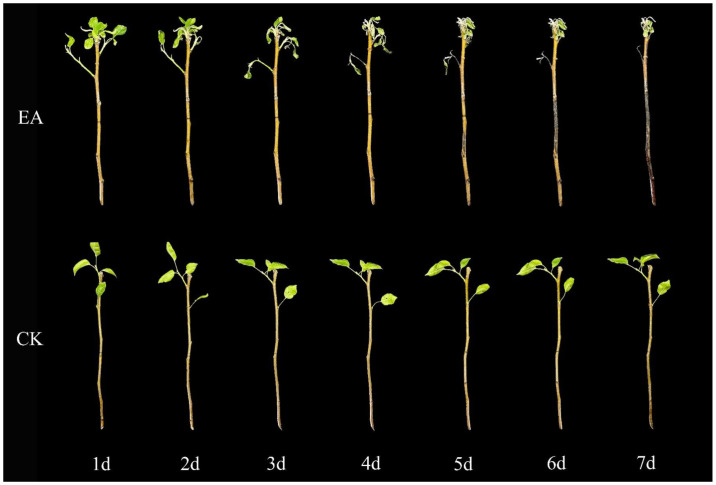
The susceptibility of Korla Xiangli branches one to seven days after inoculation with fire blight. Note: EA represents the treatment consisting of a single inoculation with *Erwinia amylovora*, and CK represents the treatment consisting of a single inoculation with clear water.

To evaluate the pathogenicity of fire blight strains affecting the Korla Xiangli, healthy leaves and twigs were inoculated with the test strains under controlled environmental conditions of 28 °C and 75% relative humidity. Significant lesions were observed at the inoculation sites on the leaves infected with the strains by the fourth day post-inoculation; this observation comes in contrast to what was observed for the sterile water control group, which exhibited no abnormalities. The pathogen demonstrated a tendency to spread along the leaf veins, leading to a progressive darkening of the leaves, which took on a brown hue (Figure 4). Similarly, the inoculated detached Korla Xiangli twigs began to develop sunken lesions as early as the third day post-inoculation. These lesions evolved into an ulcer-like appearance, transitioning in color from brown to black over time. Eventually, the affected twigs adopted a characteristic ‘cane’ shape, while the leaves on these twigs began to wilt and drop off starting on the third day. Conversely, the twigs in the sterile water control group showed no symptomatology (Figure 5).

### 2.4. The Antibacterial Effects of the Three Antagonistic Bacteria on Agar Plates

**Figure 6:**
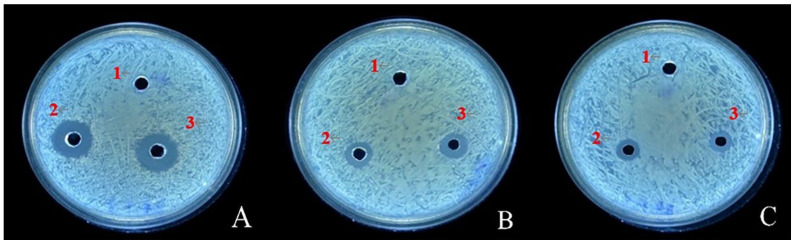
Antibacterial effects of the three antagonistic bacteria on agar plates. Note: (**A**) represents Mg-7, (**B**) represents Rt-10, and (**C**) represents Rt-11. Hole 1 is the CK control (sterile water), while Holes 2 and 3 correspond to the respective antagonistic bacteria.

The inhibitory effects of Mg-7, RT-10, and RT-11 were evaluated using the agar diffusion method, wherein the diameter of the inhibition zone indicates the antibacterial capacity of the treatments. The agar plates were evenly inoculated with the fire blight pathogen. No inhibition zone was observed around the control group (Hole 1), which contained only sterile water. In contrast, distinct inhibition zones with clear boundaries appeared around holes 7-A-2 and 7-A-3 (Figure 6). The diameter of the inhibition zone for Mg-7 was 16.1 mm (Table 1), which is significantly larger than the diameters for RT-10 (11.04 mm) and RT-11 (9.76 mm).

### 2.5. Evaluation of the Indoor Control Efficacy of Three Antagonistic Bacteria Against Fire Blight Disease

#### 2.5.1. Evaluation of the Control Effect of Detached Leaves

**Figure 7:**
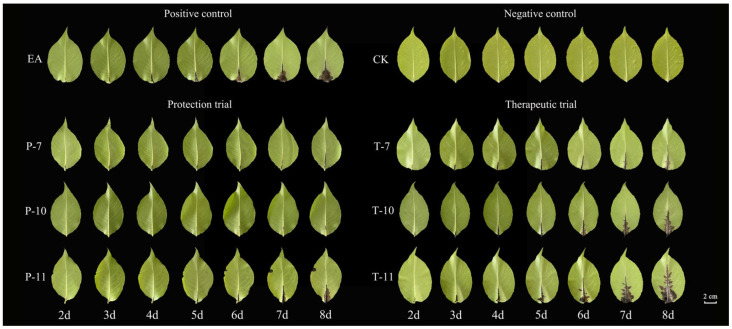
Changes in the detached leaves from days two to eight in the therapeutic and protective experiments. Note: T-7, T-10, and T-11 represent the therapeutic tests conducted using *Leuconostoc mesenteroides*, *Alcaligenes faecalis*, and *Bacillus siamensis,* respectively, while P-7, P-10, and P-11 represent the protective tests conducted using *Leuconostoc mesenteroides*, *Alcaligenes faecalis*, and *Bacillus siamensis*, respectively. EA was used for the treatment of *Erwinia amylovora* only via inoculation, while CK represents the treatment consisting of a single inoculation with clear water.

Three days post-inoculation with EA, the leaves treated with the experimental agent (EA) began to exhibit pronounced disease spots, which progressively spread along the main vein over time. In contrast, the leaves subjected to the control treatment (CK) displayed no discernible abnormalities (Figure 7). The results of the protective experiment demonstrated that the disease index for the Rt-10 treatment on the second day was significantly lower (8.91) than that for the EA treatment (14.13) (Table 2). By the eighth day, the disease index for Rt-10 rose to only 21.81, representing a notable reduction compared to the other treatments, with a control efficacy reaching up to 76.96%.

In the therapeutic experiment, although the leaves subjected to the Mg-7 treatment began to show evident disease spots by the fourth day, the treatment effectively mitigated the progression of the disease from the sixth day onward relative to the Rt-10 and Rt-11 treatments. By the eighth day, the disease index for Mg-7 was recorded to be 24.55, indicating a control efficacy of 60.39%.

#### 2.5.2. Evaluation of the Control Effect of Detached Branches

**Figure 8:**
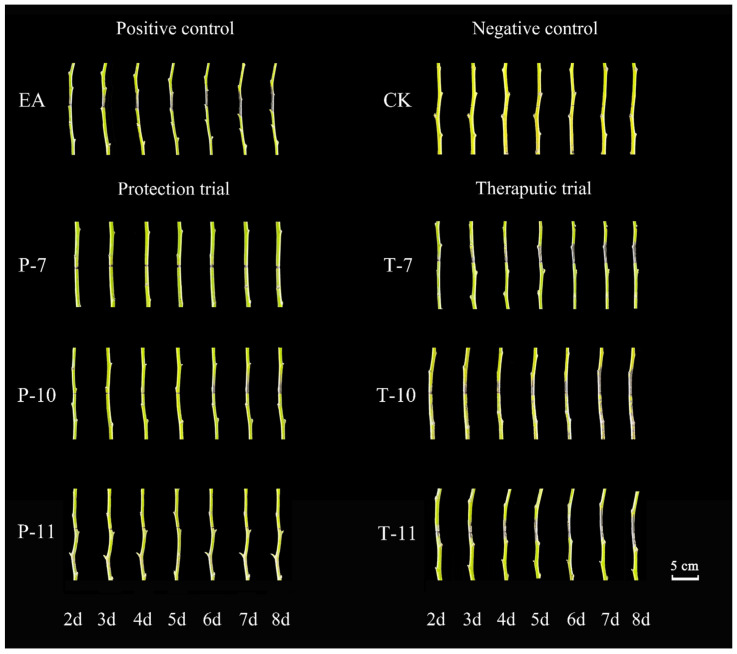
Changes in detached branches from days two to eight in the therapeutic and protective experiments. Note: T-7, T-10, and T-11 represent the therapeutic tests conducted using *Leuconostoc mesenteroides*, *Alcaligenes faecalis*, and *Bacillus siamensis*, respectively, while P-7, P-10, and P-11 represent the protective tests conducted using *Leuconostoc mesenteroides*, *Alcaligenes faecalis*, and *Bacillus siamensis*, respectively. EA was used for the treatment of *Erwinia amylovora* only via inoculation, while CK represents the treatment consisting of a single inoculation with clear water.

The disease exhibited a rapid progression on the Korla Xiangli branches treated with EA, characterized by the emergence of pronounced black disease spots that began spreading from the inoculation point as early as day three (Figure 8). By day eight, the disease index increased to 26.34. In stark contrast, no disease spots or bacterial exudates were observed around the inoculation site in the CK treatment. The findings of the protective experiment (Table 3) indicated that while disease spots began to manifest at the inoculation point in the Rt-11 treatment starting on day four, their spread remained minimal. On day eight, the disease index for Rt-11 was recorded to be 12.76, significantly lower than the values of the other treatments, demonstrating a control efficacy of 86.27%.

In the therapeutic experiment, the leaves subjected to the Mg-7 treatment exhibited noticeable disease spots by the third day, and these continued to proliferate. However, compared to the Rt-10 and Rt-11 treatments, the rate of spread for Mg-7 began to decelerate significantly starting on the fifth day. By day eight, the disease index for Mg-7 reached 19.34, which was considerably lower than that for both Rt-10 and Rt-11, yielding a control effect of 45.90%.

### 2.6. Changes in Enzyme Activity and MDA Content in Detached Branches and Leaves After Treatment with Three Types of Antagonistic Bacteria

#### 2.6.1. The Changes of Enzyme Activity and MDA Content of Detached Leaves

**Figure 9:**
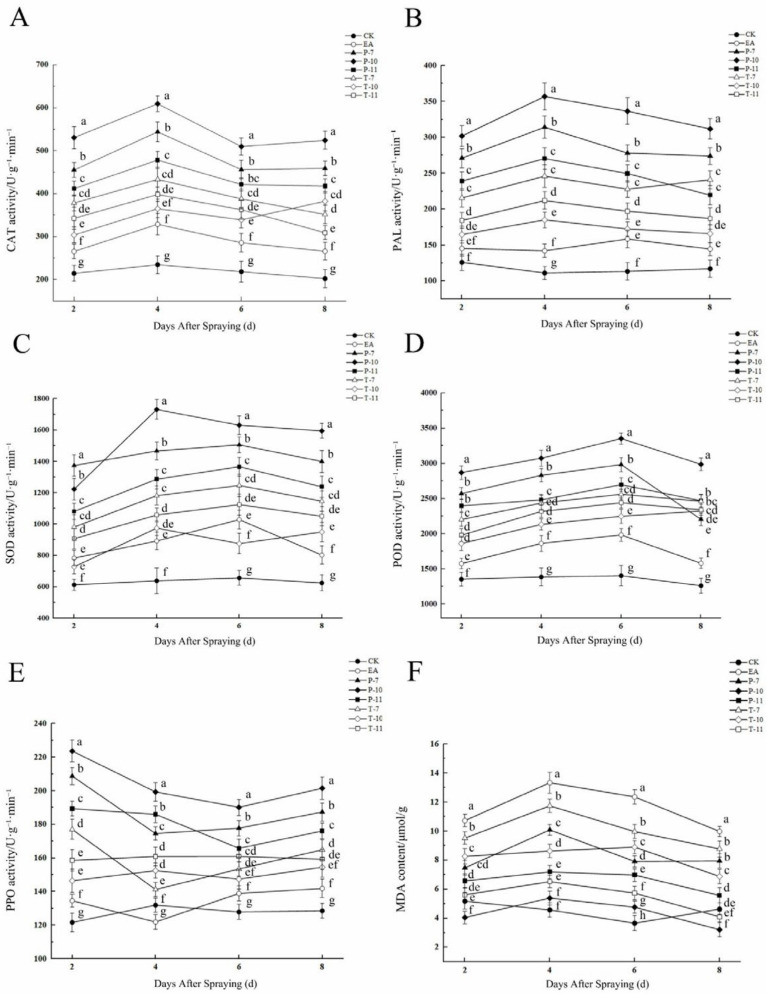
Changes in enzyme activity and MDA content in the detached leaves from days two to eight. Note: (**A**) The changes in the CAT activity of the detached leaves from day two to day eight after treatment with three antagonistic bacteria; (**B**) the changes in PAL activity from day two to day eight; (**C**) the changes in SOD activity from day two to day eight; (**D**) the changes in POD activity from day two to day eight; (**E**) the changes in PPO activity from day two to day eight; (**F**) the changes in MDA content from day two to day eight. T-7, T-10, and, T-11 represent the therapeutic tests conducted using *Leuconostoc mesenteroides*, *Alcaligenes faecalis*, and *Bacillus siamensis*, respectively, while P-7, P-10, and P-11 represent the protective tests conducted using *Leuconostoc mesenteroides*, *Alcaligenes faecalis*, and *Bacillus siamensis*, respectively. EA was used for the treatment of *Erwinia amylovora* only via inoculation, while CK represents the treatment consisting of a single inoculation with clear water. Similar letter designations among the means indicate there are no statistically significant differences (Tukey *p* < 0.05).

Infection with the fire blight pathogen induced significant dynamic changes in the activities of plant defense enzymes; among them, the activities of PAL, POD, and SOD showed a typical defense pattern of initial induction and then inhibition (Figure 9; Supplementary Tables S1–S6). The P-10 treatment had the strongest defense activation effect. Its PAL activity reached a peak on the fourth day (356.89 U·g^−1^·min^−1^); this value was significantly, i.e., 60.2%, higher compared with that for the EA control (Figure 9B). In terms of oxidative stress, the P-10 treatment effectively reduced the degree of membrane lipid peroxidation. The MDA content decreased to 3.21 μmol/g on the eighth day, and this value is only 24.1% of the peak value of the EA treatment (13.34 μmol/g) (Figure 9F). There were differences in the responses of the antioxidant enzyme system to pathogen stress: the POD activity in the P-7 treatment was the highest (2983.33 U·g^−1^·min^−1^), at 33.5% higher than that in the EA treatment (Figure 9D). The SOD activity reached its peak on the fourth and sixth days under the treatment P-10 and P-7 treatments (1731.39 and 1505.87 U·g^−1^·min^−1^), respectively, with increases of 48.5% and 31.7%, respectively (Figure 9C). The CAT activity presented a two-phase response characteristic. The T-10 treatment showed a secondary peak on the eighth day. Among the treatments, the activity of the T-7 treatment (432.96 U·g^−1^·min^−1^) was significantly higher than that of the EA treatment (24.1%). A key enzyme for lignification, the activity of PPO rapidly increased to 223.7 U·g^−1^·min^−1^ on the second day of P-10 treatment, a value 39.8% higher than that for the EA treatment, indicating the activation of the early defense mechanism.

#### 2.6.2. The Changes of Enzyme Activity and MDA Content of Detached Branches

**Figure 10:**
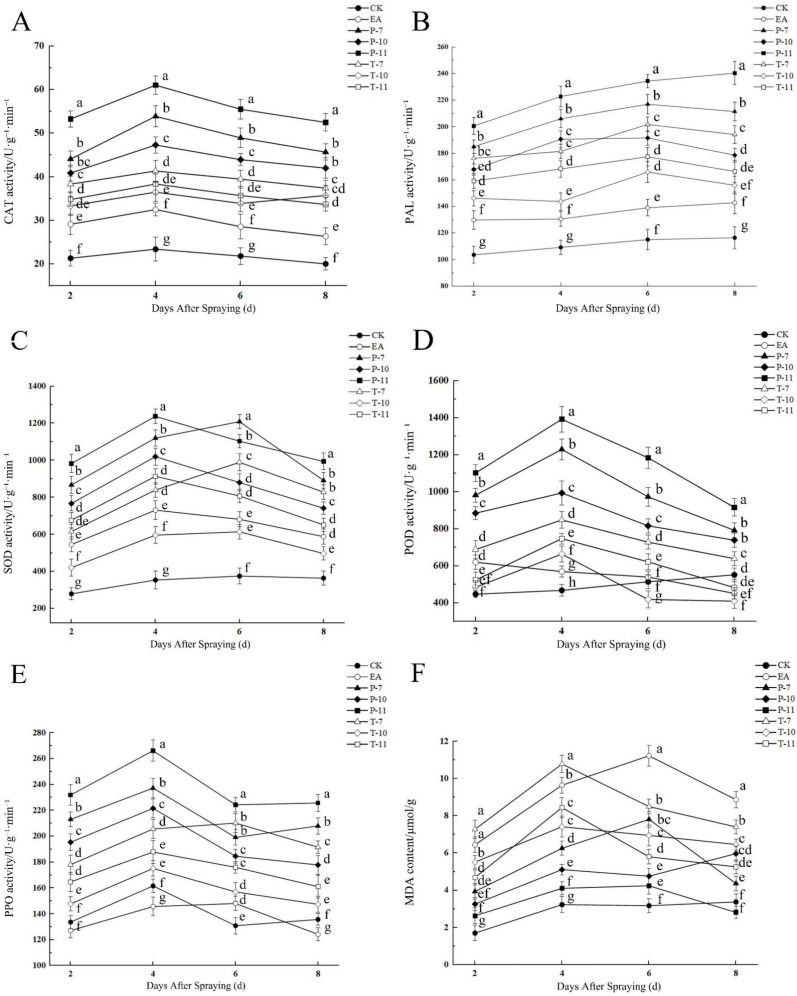
Changes in enzyme activity and MDA content in the detached branches from days two to eight. Note: (**A**) The changes in the CAT activity of the detached leaves from day two to day eight after treatment with three antagonistic bacteria; (**B**) the changes in PAL activity from day two to day eight; (**C**) the changes in SOD activity from day two to day eight; (**D**) the changes in POD activity from day two to day eight; (**E**) the changes in PPO activity from day two to day eight; (**F**) the changes in MDA content from day two to day eight. T-7, T-10, and, T-11 represent the therapeutic tests conducted using *Leuconostoc mesenteroides*, *Alcaligenes faecalis*, and *Bacillus siamensis*, respectively, while P-7, P-10, and P-11 represent the protective tests conducted using *Leuconostoc mesenteroides*, *Alcaligenes faecalis*, and *Bacillus siamensis*, respectively. EA was used for the treatment of *Erwinia amylovora* only via inoculation, while CK represents the treatment consisting of a single inoculation with clear water. Similar letter designations among the means indicate there are no statistically significant differences (Tukey *p* < 0.05).

Infection with the fire blight pathogen (EA treatment) induced significant oxidative stress in the plants, with the MDA content reaching its peak (11.23 μmol/g) on the sixth day, indicating the most severe membrane lipid peroxidation damage (Figure 10; Supplementary Tables S7–S12). In contrast, treatment with antagonistic bacteria (P-11) effectively alleviated oxidative damage by rapidly activating the antioxidant defense system, inducing peaks in the activities of CAT (61.02 U·g^−1^·min^−1^), POD (1392.04 U·g^−1^·min^−1^), SOD (1237.37 U·g^−1^·min^−1^), and PPO (266.23 U·g^−1^·min^−1^) on the fourth day. The T-10 treatment elicited a sustained defense response, with enzyme activities significantly increased by 35.7% compared to that for the leaves subjected to EA treatment on the eighth day (Figure 10A). The P-7 treatment led to the maximum increase in POD activity (53.7%) on the fourth day and maintained a relatively high level on the sixth day (Figure 10D). Notably, the P-11 treatment maintained a continuous increase in PAL activity (240.39 U·g^−1^·min^−1^, a 40.6% increase compared to EA) during the late stage of pathogen infection (the eighth day), suggesting that it may enhance plant disease resistance by strengthening the phenylpropanoid metabolism pathway (Figure 10B). In contrast, the PPO activity of the plants subjected to the T-7 treatment reached its peak (210.24 U·g^−1^·min^−1^, a 29.6% increase compared to EA) on the sixth day but declined later (Figure 10E). This indicates that antagonistic bacterium treatments (such as P-11 and P-7) can activate this plant’s antioxidant and secondary metabolic defense systems earlier and more persistently, thereby reducing pathogen-induced oxidative damage, while some treatments (such as T-10) have delayed but sustained defense enhancement effects.

### 2.7. Evaluation of the Outdoor Control Efficacy of Three Antagonistic Bacteria Against Fire Blight Disease

Following the application of antagonistic bacterial suspensions in outdoor orchards harboring plants with high susceptibility to the investigated disease, distinct antibacterial effects were recorded. At 15 days post-application, the Mg-7-100 treatment, after being diluted 100 times, exhibited a disease index of only 7.10. This represents a significant reduction compared to the other treatments, achieving a control efficacy of 61.33% (Table 4). At the 30-day mark after spraying, the disease index was 8.64, reflecting a modest increase of 1.54 from the 15-day measurement; however, the control efficacy improved to 63.17%. In a scenario where the solution was diluted 300 times, the Rt-11-300 treatment resulted in a disease index of 14.81, yielding a control efficacy of 43.68%. Notably, both the Mg-7 and Rt-10 treatments demonstrated a decreasing trend in terms of control efficacy as the dilution concentration was reduced. Conversely, for the Rt-11 treatment, dilution from 200 times to 300 times resulted in a slight increase of 1.57 in the disease index; nonetheless, this alteration led to a 6.72% improvement in control efficacy.

## 3. Discussion

The increasing prevalence of fire blight disease presents a significant challenge for the Korla Xiangli industry. Biological control has emerged as a critical strategy for mitigating plant diseases while also reducing environmental pollution and the reliance on chemical pesticides. This approach depends heavily on the identification and utilization of efficient antagonistic bacteria [18]. As a result of ongoing research into beneficial microorganisms, the integration of biocontrol agents in plant disease management has gained considerable traction. Researchers have successfully isolated and characterized antagonistic microbial strains, including *Pseudomonas* [19], *Bacillus* [20], *Agrobacterium* [21], *Trichoderma* [22], and *Saccharomyces* [23], which exhibit significant inhibitory effects against various plant pathogens.

In this study, we examined three antagonistic bacterial strains (Mg-7, Rt-10, and Rt-11) belonging to the genera *Lactobacillus*, *Rhodococcus*, and *Bacillus*, respectively. Notably, Mg-7 demonstrated a marked inhibition zone on agar plates, with antibacterial properties similar to those exhibited by *Pseudomonas graminis 49M*, as reported by Mikiciński et al. [24]. In the protection experiment conducted on Korla Xiangli leaves in this study, the control efficacy of Rt-10 was 76.96%, which was higher than that of the *Streptomyces* sp. JCK-8055 strain against fire blight on apples (69.37%). However, the Korla Xiangli is a variety that is highly susceptible to fire blight, and we found that exhibited greater control efficacy of Rt-10 on Korla Xiangli, a more susceptible host than JCK-8055 on apples, which represent a moderately resistant host. This further validates the more outstanding control efficacy of Rt-10 against fire blight [25]. When the application concentration of Rt-11 was lower than that of *Bacillus velezensis* JE4, its control efficacy against fire blight on Korla Xiangli branches still reached 86.27%, a value significantly higher than that of JE4 (74%), which indicates that Rt-11 can maintain a high level of biocontrol efficacy and stronger colonization ability even under low-dose conditions [26]. *Bacillus amyloliquefaciens* MB40 isolated by Shemshura O et al. [27] can destroy the integrity of pathogenic bacterial membranes or interfere with quorum sensing by decomposing volatile organic compounds (2,3-butanedione), achieving an inhibition efficiency of 90.6% against *Erwinia amylovora*. In contrast, in our study, Mg-7, Rt-10, and Rt-11 mainly manifested their effects when the plants were infected by pathogenic bacteria, as the antagonistic bacteria reduce the accumulation of reactive oxygen species (ROS) through antioxidant enzymes, thereby eliminating and alleviating the oxidative damage pathogenic bacteria inflict on plants.

Moreover, Esteban et al. [3] documented that *Pseudomonas fluorescens* UV-30 exhibited a high control efficacy of 89% against fire blight on day three of their experiment, and this value diminished to 67% by day five. These findings align with our results, showing a gradual decline in the antibacterial efficacy of the antagonistic bacteria from day four to day six. Both curative and protective control efficacy evaluations underscore the superior effectiveness of protective measures over curative interventions. This observation corroborates the findings reported by Cui et al. [28], who revealed a higher disease index in curative experiments (2.27) compared to protective experiments (1.78) involving the use of *P. megaterium* strain KD7 in controlled indoor settings against fire blight. Although the three antagonistic bacteria examined in this study have not been previously reported for use in fire blight control, they exhibited considerable biocontrol potential in in vitro experiments, achieving maximum control efficacies of 76.96% on leaves and 86.27% on twigs. Nevertheless, further comprehensive research is essential for optimizing their fermentation conditions and elucidating the underlying mechanisms of disease prevention and antibacterial activity.

Induced resistance is the key mechanism of the biological control of plant diseases. The changes in the activity of defense enzymes and the content of MDA are the key factors used to evaluate plant disease resistance. Volatile organic compounds produced by different antagonistic bacteria strains can trigger the salicylic acid (SA) and jasmonic acid (JA) signaling pathways, thereby potentiating a plant’s resistance to pathogenic bacteria and enhancing its defense against biological stress [29]. A genome-wide analysis of the AhBAG gene family in peanut conducted by Zhao et al. [30] revealed the crucial roles of these genes in plant defense signaling pathways. Their study demonstrated that specific AhBAG members were significantly upregulated upon infection with a pathogen and exhibited crosstalk with both the SA and JA signaling pathways. Ilham B et al. [31] used *Arabidopsis thaliana* as a subject to explore the effects of *Bacillus amyloliquefaciens* (I3) and *Trichoderma harzianum* (A) on inducing plant systemic resistance. Their study revealed that these strains (I3 and A) could significantly activate the SA and JA signaling pathways in *Arabidopsis thaliana*, efficiently triggering the plant’s defense mechanisms, and the induction effect was superior to that of chemical elicitors.

Typically, when infected or under stress, plant cells initiate defense responses, leading to the activation of enzymes such as POD, CAT, SOD, and PPO to reduce the accumulation of reactive oxygen species (ROS) and minimize cell damage [32]. MDA is one of the most important products of lipid peroxidation in cell membranes. Its occurrence aggravates the damage inflicted on cell membrane lipids. By detecting its content, the degree of lipid peroxidation in cell membranes can be ascertained, in turn allowing the degree of damage dealt to the membrane system to be evaluated [33]. In this study, the MDA content of the leaves and branches treated with Mg-7 was significantly lower than that of the leaves and branches inoculated with *Erwinia amylovora* (EA treatment), indicating that the Mg-7 treatment effectively alleviated the damage dealt by *Erwinia amylovora* to plant cell membrane lipids. Furthermore, studies have shown that the activities of PAL and CAT usually present a dynamic trend of first increasing and then decreasing, which is positively correlated with the disease resistance of plants [34]. This trend is consistent with our research results. The activities of PAL and CAT in the branches and leaves treated with Mg-7 were significantly higher than those in the branches and leaves treated with EA. The increase in CAT activity observed indicates that hydrogen peroxide could be removed more effectively under the Mg-7 treatment, protecting cells from oxidative damage. The increase in PAL indicates that the induction of the activity of antagonistic bacteria can lead to the acceleration of the phenylpropane metabolic pathway, allow the synthesis of lignin and other resistance substances, and enhance the defensive ability of plants [35]. POD and SOD are the key enzymes involved in the mechanisms of plant scavenging of reactive oxygen species. When plants are under stress conditions, they produce a large quantity of reactive oxygen species. Our experiment shows that the activities of POD and SOD in the plants treated with P-7 and P-11 were both better than those in the plants treated with EA inoculation alone, indicating that the treatments with the antagonistic bacteria Mg-7 and Rt-11 promoted increased SOD activity. This method effectively eliminates reactive oxygen species in plants, reducing the oxidative damage that pathogenic bacteria inflict on plant cells. In addition, when the activity of antagonistic bacteria is induced, POD can promote the synthesis of lignin, enhance the strength of the cell wall, prevent the invasion of pathogenic bacteria, and enhance the ability of plant cells to recover from ROS-induced damage, confirming the discovery made by Li et al. [36]. PPO activity is an indicator of plant disease resistance. In this study, the PPO activity of the Korla Xiangli leaves treated with Rt-10 was significantly higher than in the leaves treated with a single inoculation of *Erwinia amylovora,* indicating that the PPO activity was increased under the Rt-10 treatment, promoting the oxidation of phenolic substances and enhancing the plants’ disease resistance [37].

Moreover, it is essential to recognize that the conditions of laboratory-based antibacterial and inoculation experiments may markedly differ from field conditions, where diverse microbial populations have distinct requirements and exhibit adaptability to environmental factors [38]. The existing literature suggests that the efficacy of antagonistic bacteria diminishes with an increasing dilution ratio [39], a finding that is consistent with our observations indicating reduced efficacy at lower concentrations. The control efficacy of 66.36% reported by Wang et al. [40] using 3% thiamethoxam water-dispersible granules against fire blight under field conditions is comparable to the 61.33% control efficacy achieved using the Mg-7-100 treatment 15 days post-application in the outdoor trial in this study. Notably, the microbial antagonists employed in this study do not induce drug resistance and demonstrate sustained efficacy (63.17%) even 30 days after application. Although the outdoor study primarily focused on phenotypic assessments, further in-depth research is essential to identify the underlying mechanisms governing their antibacterial actions in the field.

## 4. Materials and Methods

### 4.1. Test Strains

The three antagonistic bacterial strains tested (Mg-7, Rt-10, and Rt-11) were obtained by our research group (Table A1). The fire blight pathogen strain was isolated from and identified in suspected fire-blight-infected branches and leaves collected from Awati Township, Korla, Xinjiang, and preserved. It was confirmed to be a highly pathogenic strain of the fire blight pathogen.

### 4.2. Experimental Site

The experimental site is located in the Korla Xiangli Orchard in Korla, Xinjiang Uyghur Autonomous Region (41°43′33″ N, 86°10′29″ E). The variety tested was the Korla Xiangli, with samples taken from trees that were approximately 15 years old, maintained using conventional water and fertilizer management practices, and exhibited consistent plant growth. For the indoor experiments, healthy, disease-free, one-year-old Korla Xiangli branches of uniform length and young leaves of the same size were selected for in vitro therapeutic and protective trials. The outdoor experiments were conducted in a high-susceptibility-to-fire-blight orchard in the same area, with tree vigor and orchard management conditions consistent with those of the in vitro trial orchard.

### 4.3. Isolation and Identification of Fire Blight

#### 4.3.1. Isolation and Purification

The suspected fire blight pathogen strain was isolated and purified using the streak plate method [41]: A 0.5 cm^2^ tissue sample was cut from the junction between healthy and diseased tissue. The processed tissue sample was then cut into small pieces and surface-disinfected with 75% ethanol for 30 s and then 2% sodium hypochlorite for 60 s. After being rinsed three times with sterile water, the sample was placed in a Petri dish and incubated in a 28 °C incubator for 48 h. After colony formation had begun, individual bacterial clones with similar colors and morphological characteristics were selected, and pure cultures were established on an NA medium, with a total of four plates. The strain was named Xea.

#### 4.3.2. Molecular Biology Verification

Referring to the method used to detect fire blight pathogens reported by Kim et al. [42], the universal primers 27F (5′ AGAGTTTGATCCTGGCTCAG 3′) and 1492R (5′ GGTTACCTTGTTACGACTT 3′) synthesized by Beijing Dingguo Changsheng Biotechnology Co., Ltd. (Urumqi, China), were used. The re-inoculated and purified single colony was used as the DNA template to prepare a 30 μL reaction system: 15 μL of Taq enzyme, 11 μL of ddH_2_O, 1 μL (10 μmol/L) of upstream primer, and 1 μL (10 μmol/L) of downstream primer. Then, a PCR amplification reaction was carried out according to the following program: initial denaturation at 94 °C for 5 min, denaturation at 94 °C for 30 s, annealing at 54 °C for 30 s, extension at 72 °C for 45 s for 35 cycles, and a final extension at 72 °C for 7 min. The amplified complete system template was sent to a biological company to be sequenced, and the obtained sequences were submitted to the NCBI database for BLAST comparison (https://blast.ncbi.nlm.nih.gov/Blast.cgi, accessed on 10 March 2025). The films were centrally disinfected.

The sequencing results were subjected to multiple sequence alignment with the relevant strains in the GenBank database using the MegAlign module available in the DNAStar software, version 11.1 package, and 13 model strains with the closest genetic relationships were screened out. Subsequently, the adjacency method in MEGA7.0 software was used to construct the phylogenetic tree, and the bootstrap value was set to 1000 repeats to evaluate the reliability of the phylogenetic tree.

#### 4.3.3. Observation of Morphological Characteristics

Referring to He et al.’s method [43], we inoculated the tested *Erwinia amylovora* (Xea) into nutrient AGAR (NA) medium plates and placed them in a constant-temperature incubator at 28 °C for isolation, purification, and culturing. The culture time was 24 to 36 h. After colonies had formed, their morphological characteristics were observed and recorded, including the colony size, color, surface texture, edge features, and growth status.

#### 4.3.4. Pathogenicity Assessment

Referring to the method described by Li et al. [44], we activated the fire blight pathogen strain on an NA medium plate, selected a single colony, and inoculated it into the NB culture medium. It was then shaken and cultured at 28 °C at 180 rpm for 24 h until the bacterial suspension reached an OD_600_ of 0.8–1.0. It was then diluted with sterile water to an OD_600_ of 0.2 (1 × 10^6^ CFU/mL) for later use.

The collected healthy detached branches and leaves of the Korla Xiangli variety were rinsed and disinfected with sterile water. (1) For leaf inoculation, 10 μL (1 × 10^6^ CFU/mL) of the fire blight pathogen suspension was injected into the petiole 0.5 cm from the base of the leaf using a needle. (2) For branch inoculation, branches approximately 20 cm in length were cut with pruning shears. A sterile scalpel was used to create a wound (0.2 cm) in the middle of each branch, and then 20 μL (1 × 10^6^ CFU/mL) of the fire blight pathogen suspension was inoculated into the wound using a microsyringe. Sterile water was used as a control. Three branches/leaves were used for each treatment, and the experiment was repeated three times. After inoculation, the samples were placed in an artificial climate chamber with a temperature of 28 °C and a relative humidity of 75% for cultivation. The incidence of disease on the branches and leaves was observed every 24 h for a total of 7 days.

### 4.4. Determination of Antibacterial Activity on Agar Plates

In reference to the method described by Zheng et al. [45], the antagonistic bacterial strains to be tested were streaked and activated on NA medium. Single colonies were selected with an inoculating loop and inoculated into an NB liquid medium, which was then shaken and cultured at 30 °C at 180 rpm for 12–18 h to obtain seed cultures. The seed cultures were inoculated into the NB liquid medium at a 5% inoculation rate and shaken and cultured at 30 °C at 180 rpm for 48 h until the OD_600_ reached 0.8–1.0 to obtain a fermentation broth. The fermentation broth was centrifuged at 4 °C at 12,000 rpm for 30 min to obtain the supernatant filtrate, which was set aside for later use.

The fire blight pathogen was diluted to an OD_600_ of 0.2 using a 10-fold dilution method. Then, 100 μL of the diluted pathogen was evenly spread on NA medium using sterilized glass beads. Three holes were evenly punched in the medium using a puncher (5 mm in diameter), and 70 μL of the antagonistic bacterial supernatant filtrate was added to each hole. The concentrations of the three antagonistic bacteria solutions were approximately 1 × 10^8^ CFU/mL, with sterile water serving as a control. Each treatment was divided into three groups, with three replicates per group. The cultures were incubated at 28 °C for 24 h, and the diameters of the inhibition zones were measured and recorded.

### 4.5. Indoor Efficacy Determination

#### 4.5.1. Detached Korla Xiangli Leaves

Referring to He et al.’s method [46], we used fully expanded, young, and healthy detached Korla Xiangli leaves. After washing the leaves with pure water, we sterilized them using ultraviolet light for 20 min. The excess petioles were trimmed to about 0.5 cm, and incisions of the same size were made on the backs of the leaves using a scalpel. Inoculations were performed at the incision sites using a microsyringe. (1) For the therapeutic trial, 10 μL OD_600_ of 0.2 (1 × 10^6^ CFU/mL) of the *Erwinia amylovora* suspension was inoculated first, followed by 10 μL OD_600_ of 0.8 (1 × 10^8^ CFU/mL) of the antagonistic bacterial suspension 24 h later; (2) for the protective trial, 10 μL OD_600_ of 0.8 (1 × 10^8^ CFU/mL) of the antagonistic bacterial suspension was inoculated first, followed by 10 μL OD_600_ of 0.2 (1 × 10^6^ CFU/mL) of the *Erwinia amylovora* suspension 24 h later. Samples inoculated with sterile water served as a control. Subsequently, moist sterile filter paper was placed in sterile Petri dishes, and a triangular support was created using sterile toothpicks to hold the leaves with their backs side up, preventing the leaves from coming into contact with water and turning brown. There were eight treatments in total, as shown in Table 1, with nine leaves per treatment and three replicates.

#### 4.5.2. Detached Korla Xiangli Branches

Detached one-year-old shoots were used and uniformly trimmed to a length of about 20 cm. Incisions of uniform size were made in the middle sections of the shoots using a scalpel, and inoculations were performed at the incision sites. The procedures for the therapeutic and protective tests conducted on the detached branches were the same as those for the leaves, but with different inoculation volumes: both the fire blight pathogen suspension and the antagonistic bacterial suspension were 20 μL. There were eight treatments in total, as shown in Table 1, with six branches per treatment and 3 replicates.

All the samples were placed in a 28 °C incubator. Since the antagonistic bacterial suspension and the fire blight pathogen suspension were inoculated 24 h apart in the therapeutic and protective trials, respectively, disease indices were recorded starting on the second day. The incidence of disease on the leaves and branches was observed and recorded every 24 h for 8 days. The symptoms of disease on the leaves and branches were observed and recorded individually for each treatment. The treatments for the therapeutic and protective trials applied to the detached branches and leaves of the Korla Xiangli are shown in Table 5.

Control efficacy (%) = (length of lesion on control leaf/branch-length of lesion on leaf/branch treated with antagonistic bacteria)/length of lesion on control leaf/branch × 100.

### 4.6. Determination of Enzyme Activity and MDA Content

Samples were taken from the second day to the eighth day during the evaluation of the control efficacy on the detached branches and leaves. The collected samples were wrapped in aluminum foil and stored in liquid nitrogen. Enzyme activity was determined according to Usha et al.’s method [47], with appropriate adjustments. The specific steps are as follows: SOD was assessed using the nitroblue tetrazolium method; POD was determined using the guaiacol method; CAT was measured using the ultraviolet absorption method; PPO was assessed using the catechol method; and PAL was detected using the ultraviolet spectrophotometric method. Additionally, MDA content was quantitatively analyzed using the thiobarbituric acid method. There were eight treatments in the experiment, and each treatment was repeated three times; the treatments for each experiment are shown in Table 4.

### 4.7. Outdoor Efficacy Evaluation

Because of the uncertainty regarding the potential harm the three antagonistic bacteria could inflict on the plants in the Korla Xiangli orchard and given that current scientific research has not fully elucidated their specific mechanisms of action, it was crucial to ensure that the probiotic effects of the antagonistic bacteria were utilized while avoiding negative impacts on the plants. Therefore, we conducted this evaluation based on the method described by Liu et al. [48], with improvements made in order to dilute the bacteria to an appropriate concentration before spraying the plants. The trial had a randomized block design with single-plant plots, with nine replicates for each treatment, totaling 90 experimental trees across 10 treatments (Table 6). The first spraying was conducted on 12 April 2024 (the early-bloom stage); the second was conducted on 23 April (the late-bloom stage); and the third was conducted on 4 May (after petal fall). A knapsack electric sprayer was used to apply the antagonistic bacterial solution uniformly and without dripping. The weather was sunny and windless during the application, and there was no precipitation within 48 h after the application. No fungicides had been applied to control diseases in this orchard prior to this trial. The trial included two data collection points: 19 May (15 days after spraying) and 3 June (30 days after spraying). During data collection, five branches were selected from the east, west, south, and north for each treatment (and marked with tags before application) to count the number of diseased branches and measure lesion length.

### 4.8. Disease Index Statistics

Based on the method described by Campbell et al. [49] (with improvements), the disease development of fire blight after in vitro inoculation was graded as follows:

Grade 0—no lesions observed;

Grade 1—a lesion whose length accounts for 1% to 5% of the total length of the inoculated young leaf or twig;

Grade 3—a lesion whose length accounts for 5.1% to 15% of the total length;

Grade 5—a lesion whose length accounts for 15.1% to 30% of the total length;

Grade 7—a lesion whose length accounts for 30.1% to 50% of the total length;

Grade 9—a lesion whose length exceeds 50.1% of the total length of the inoculated young leaf or twig.

The disease index (DI), incidence rate, and control efficacy were calculated using the following formulas:

DI = [∑ (number of diseased branches in each grade × representative value of the grade)/(total number of diseased branches × representative value of the highest grade)] × 100; incidence rate = (number of diseased branches/total number of branches) × 100%; control efficacy = (disease index of the control group − disease index of the treated group)/disease index of the control group × 100%.

### 4.9. Data Processing

All experimental data were statistically analyzed using Excel 2016 and SPSS 22.0 software. Duncan’s new multiple-range test (*p* < 0.05) was used for the analysis of significant differences. Origin 2021 was used to plot the graphs.

## 5. Conclusions

In this study, three antagonistic bacterial strains demonstrated substantial phytoprotective efficacy in both therapeutic and preventive in vitro applications to Korla Xiangli leaves and twigs. These strains significantly reduced plant disease indices while enhancing the activities of enzymes such as CAT and PPO. A comparative analysis revealed a concomitant decrease in MDA content relative to pathogen-exclusive inoculations. Notably, the preventive applications exhibited a disease suppression capacity superior to that of the therapeutic interventions. Outdoor trials showed that spraying the antagonistic bacteria improved the efficacy of control on the plants and reduced the disease indices in the field. Based on the performance of the three antagonistic bacteria, Mg-7 showed more significant control effects against fire blight on the pear trees in vitro, with the smallest lesion expansion area on Korla Xiangli twigs and leaves. In the outdoor trials, Mg-7 outperformed Rt-10 and Rt-11 in terms of overall performance and exhibited good stability. Therefore, Mg-7 has potential research value in the context of biological control.

## Figures and Tables

**Table 1:** The antibacterial effects of the three antagonistic bacteria on the plates.

Different Antagonistic Treatment	Diameter of the Bacteriostatic Circle (mm)
Mg-7	16.10 ± 0.19 a
Rt-10	11.04 ± 0.18 b
Rt-11	9.76 ± 0.12 c

Note: Mg-7, Rt-10, and Rt-11 represent the therapeutic tests conducted using *Leuconostoc mesenteroides*, *Alcaligenes faecalis*, and *Bacillus siamensis,* respectively. Similar letter designations among the means indicate there are no statistically significant differences (Tukey *p* < 0.05). The data were expressed in the following form: average value ± standard deviations.

**Table 2:** Disease index and control efficacy of the detached leaves.

	2d	3d	4d	5d	6d	7d	8d	
Treatment Group	Disease Index	Disease Index	Disease Index	Disease Index	Disease Index	Disease Index	Disease Index	Control Effect
T-7	12.21 ± 0.72 b	14.95 ± 0.95 cd	18.24 ± 0.73 b	20.44 ± 0.28 cd	22.91 ± 0.27 c	24.28 ± 0.83 d	24.55 ± 0.82 c	60.39 ± 0.44 c
T-10	13.19 ± 0.27 b	16.59 ± 0.48 b	19.88 ± 0.28 a	21.99 ± 0.48 b	24.55 ± 0.99 b	27.20 ± 0.48 b	27.85 ± 0.55 b	45.95 ± 0.74 e
T-11	13.07 ± 0.73 ab	14.95 ± 0.24 c	19.61 ± 0.27 a	20.71 ± 0.28 c	23.28 ± 0.47 bc	26.02 ± 0.47 c	26.87 ± 0.48 b	51.92 ± 0.71 d
P-7	10.29 ± 0.48 c	12.75 ± 1.43 de	16.32 ± 0.27 c	19.34 ± 0.82 d	19.61 ± 0.27 e	22.73 ± 1.99 d	24.10 ± 0.91 c	63.30 ± 0.52 b
P-10	8.91 ± 0.73 d	11.66 ± 0.95 e	13.03 ± 0.55 d	18.29 ± 0.29 e	19.06 ± 0.55 e	20.44 ± 1.26 e	21.81 ± 0.96 d	76.96 ± 0.46 a
P-11	10.84 ± 0.48 c	13.88 ± 0.26 d	16.60 ± 0.55 c	18.83 ± 1.98 de	20.99 ± 0.48 d	23.81 ± 0.47 d	25.12 ± 0.95 c	59.27 ± 0.88 c
EA	14.13 ± 0.54 a	18.24 ± 0.48 a	20.44 ± 0.73 a	23.73 ± 0.99 a	27.29 ± 0.28 a	30.31 ± 0.27 a	32.96 ± 0.27 a	—

Note: T-7, T-10, and T-11 represent the therapeutic tests conducted using *Leuconostoc mesenteroides*, *Alcaligenes faecalis*, and *Bacillus siamensis*, respectively, while P-7, P-10, and P-11 represent the protective tests conducted using *Leuconostoc mesenteroides*, *Alcaligenes faecalis*, and *Bacillus siamensis*, respectively. EA was used for the treatment of *Erwinia amylovora* only via inoculation. Similar letter designations among the means indicate there are no statistically significant differences (Tukey *p* < 0.05).

**Table 3:** Disease index and control efficacy of the detached branches.

	2d	3d	4d	5d	6d	7d	8d	
Treatment Group	Disease Index	Disease Index	Disease Index	Disease Index	Disease Index	Disease Index	Disease Index	Control Effect
T-7	5.76 ± 0.41 c	10.29 ± 0.41 c	11.52 ± 0.42 c	13.17 ± 0.44 c	16.87 ± 0.41 c	18.93 ± 0.41 c	19.34 ± 0.31 c	45.90 ± 0.63 d
T-10	8.23 ± 0.43 b	11.93 ± 0.43 b	13.99 ± 1.09 b	17.28 ± 0.71 b	19.34 ± 0.51 b	20.16 ± 0.43 b	21.81 ± 0.41 b	34.64 ± 0.26 f
T-11	7.82 ± 0.41 b	11.52 ± 0.42 b	14.40 ± 0.45 b	17.69 ± 0.42 b	18.93 ± 0.41 b	19.34 ± 0.42 bc	22.22 ± 0.71 b	37.23 ± 0.36 e
P-7	4.94 ± 0.71 cd	6.47 ± 0.30 d	10.28 ± 0.82 cd	11.52 ± 0.41 d	11.94 ± 0.46 de	12.76 ± 0.41 e	16.87 ± 0.41 e	80.94 ± 0.37 b
P-10	5.76 ± 0.82 cd	6.99 ± 0.82 d	10.29 ± 0.41 d	12.35 ± 0.71 cd	12.76 ± 0.41 d	16.05 ± 0.71 d	18.11 ± 0.42 d	55.70 ± 0.62 c
P-11	4.52 ± 0.42 d	5.35 ± 0.45 e	8.08 ± 0.34 e	10.29 ± 0.82 de	11.52 ± 0.32 e	12.35 ± 0.71 e	12.76 ± 1.09 f	86.27 ± 0.28 a
EA	10.22 ± 0.45 a	15.22 ± 0.41 a	17.69 ± 0.41 a	19.34 ± 0.41 a	21.72 ± 0.83 a	23.87 ± 0.41 a	26.34 ± 0.52 a	—

Note: T-7, T-10, and T-11 represent the therapeutic tests conducted using *Leuconostoc mesenteroides*, *Alcaligenes faecalis*, and *Bacillus siamensis*, respectively, while P-7, P-10, and P-11 represent the protective tests conducted using *Leuconostoc mesenteroides*, *Alcaligenes faecalis*, and *Bacillus siamensis*, respectively. EA was used for the treatment of *Erwinia amylovora* only via inoculation. Similar letter designations among the means indicate there are no statistically significant differences (Tukey *p* < 0.05).

**Table 4:** Disease index and control efficacy statistics after antagonistic bacteria were sprayed in the field.

Day	15d		30d	
Treatment Group	Disease Index	Control Effect (%)	Disease Index	Control Effect (%)
Mg-7-100	7.10 ± 0.81 e	61.33 ± 3.85 a	8.64 ± 1.12 f	63.17 ± 4.72 a
Mg-7-200	10.80 ± 0.82 d	39.65 ± 4.56 c	12.96 ± 0.53 d	44.84 ± 2.27 b
Mg-7-300	12.04 ± 0.53 c	32.76 ± 2.98 d	15.43 ± 0.31 bc	34.34 ± 1.32 cd
Rt-10-100	10.19 ± 0.51 d	43.09 ± 2.98 c	12.35 ± 1.11 d	47.46 ± 4.73 b
Rt-10-200	12.35 ± 0.82 c	31.02 ± 4.56 d	15.12 ± 0.61 bc	35.64 ± 2.62 c
Rt-10-300	12.96 ± 1.41 c	27.60 ± 7.90 de	16.36 ± 0.81 b	30.40 ± 3.48 d
Rt-11-100	8.34 ± 1.43 e	53.43 ± 3.90 b	11.11 ± 1.61 de	52.72 ± 6.83 ab
Rt-11-200	11.11 ± 1.41 cd	37.91 ± 7.91 c	13.24 ± 0.82 d	36.96 ± 2.27 c
Rt-11-300	14.2 ± 1.11 b	20.69 ± 6.21 e	14.81 ± 0.53 c	43.68 ± 3.50 b
CK	17.90 ± 0.81 a	—	23.46 ± 1.72 a	—

Note: Mg-7, Rt-10, and Rt-11 denote the *Leuconostoc mesenteroides*, *Alcaligenes faecalis*, and *Bacillus siamensis* treatments, respectively; 100, 200, and 300 are the dilution multiples, respectively, and CK denotes the treatment that involved spraying clear water. Similar letter designations among the means indicate there are no statistically significant differences (Tukey *p* < 0.05).

**Table 5:** Treatment names and methods used in indoor efficacy determination.

Treatment Name	Treatment Method
Ea	Single inoculation with *Erwinia amylovora* (positive control)
CK	Single inoculation with sterile water (negative control)
T-7	Inoculation with the *Erwinia amylovora* first, followed by Mg-7
T-10	Inoculation with the *Erwinia amylovora* first, followed by Rt-10
T-11	Inoculation with the *Erwinia amylovora* first, followed by Rt-11
P-7	Inoculation with Mg-7 first, followed by *Erwinia amylovora*
P-10	Inoculation with Rt-10 first, followed by *Erwinia amylovora*
P-11	Inoculation with Rt-11 first, followed by *Erwinia amylovora*

Note: Mg-7 (*Leuconostoc mesenteroides*), RT-10 (*Alcaligenes faecalis*), and RT-11 (*Bacillus siamensis*). The same applies below.

**Table 6:** Treatment names and methods in the outdoor efficacy determination experiment.

Treatment Name	Treatment Method
CK	Negative control
Mg-7-100	Mg-7 diluted 100-fold
Mg-7-200	Mg-7 diluted 200-fold
Mg-7-300	Mg-7 diluted 300-fold
RT-10-100	RT-10 diluted 100-fold
RT-10-200	RT-10 diluted 200-fold
RT-10-300	RT-10 diluted 300-fold
RT-11-100	RT-11 diluted 100-fold
RT-11-200	RT-11 diluted 200-fold
RT-11-300	RT-11 diluted 300-fold

Note: The dilution ratios of the bacterial suspensions were 100, 200, and 300 times, respectively.

## Data Availability

Data are contained within the article.

## Supplementary Materials

The following supporting information can be downloaded at: https://www.mdpi.com/article/10.3390/ijms26094438/s1.

## Abbreviations

The following abbreviations are used in this manuscript:

Mg-7	*Leuconostoc mesenteroides*
Rt-10	*Alcaligenes faecalis*
Rt-11	*Bacillus siamensis*
POD	peroxidase
CAT	catalase
SOD	superoxide dismutase
PPO	polyphenol oxidase
PAL	phenylalanine ammonia-lyase
MDA	malondialdehyde
EA	Single inoculation with *Erwinia amylovora* (positive control)
CK	Single inoculation with sterile water (negative control)
T-7	Inoculate with the *Erwinia amylovora* first, followed by Mg-7
T-10	Inoculate with the *Erwinia amylovora* first, followed by Rt-10
T-11	Inoculate with the *Erwinia amylovora* first, followed by Rt-11
P-7	Inoculate with Mg-7 first, followed by *Erwinia amylovora*
P-10	Inoculate with Rt-10 first, followed by *Erwinia amylovora*
P-11	Inoculate with Rt-11 first, followed by *Erwinia amylovora*
Mg-7-100	Mg-7 diluted 100-fold
Mg-7-200	Mg-7 diluted 200-fold
Mg-7-300	Mg-7 diluted 300-fold
RT-10-100	RT-10 diluted 100-fold
RT-10-200	RT-10 diluted 200-fold
RT-10-300	RT-10 diluted 300-fold
RT-11-100	RT-11 diluted 100-fold
RT-11-200	RT-11 diluted 200-fold
RT-11-300	RT-11 diluted 300-fold

## Appendix A

**Table A1:** Fire blight fungus and three antagonistic bacteria sequences.

Strain	Sequence
*Leuconostoc mesenteroides* (Mg-7)	GTTGGTTCCTTGCCCTTAAGAACGGCTCCTTCCTAAAAGGTTAGGCCACCGGCTTTGGGCATTACAAACTCCCATGGTGTGACGGGCGGTGTGTACAAGACCCGGGAACGTATTCACCGCGGCGTGCTGATCCGCGATTACTAGCGATTCCGACTTCATGTAGTCGAGTTGCAGACTACAATCCGAACTGAGACGTACTTTAAGAGATTAGCTCACCCTCGCGGGTTGGCAACTCGTTGTATACGCCATTGTAGCACGTGTGTAGCCCAGGTCATAAGGGGCATGATGATCTGACGTCGTCCCCGCCTTCCTCCGGTTTGTCACCGGCAGTCTCGCTAGAGTGCCCATCTGAATGCTGGCAACTAACAATAAGGGTTGCGCTCGTTGCGGGACTTAACCCAACATCTCACGACACGAGCTGACGACGACCATGCACCACCTGTCACTTTGTCTCCGAAGAGAACACTTCTATCTCTAAAAGCTTCAAAGGATGTCAAGACCTGGTAAGGTTCTTCGCGTTGCTTCGAATTAAACCACATGCTCCACCGCTTGTGCGGGTTCCCGTCGTTTTCTTTGAGTTTTTTCTTTTTGGTCGTACTCCCCATGTGGAACACTTTTCGCGTTAGCTTCGGCACTAAGAGGCGGAAACCTCCTAATTTCTATTGTTTCATCTTTTATTGTGTGTACTACTTGGTTTATCTTTTTTTTTGTTACGATATTTATCCTTTCTAGCCTCAACGTCTGTTTGCTGTCCCTGTATCTCCGCCCTTCTCCACTGTCGGTTCTTCCCATATATCTACGCATTCCCCCCGGCTACACATGGGAATTCCACTTTACCCTCCA
*Alcaligenes faecalis* (Rt-10)	TACGGTTAGGCTACCTACTTCTGGTGAAACCCACTCCCATGGTGTGACGGGCGGTGTGTACAAGACCCGGGAACGTATTCACCGCGACATTCTGATCCGCGATTACTAGCGATTCCGACTTCACGCAGTCGAGTTGCAGACTGCGATCCGGACTACGATCGGGTTTCTGAGATTGGCTCCCCCTCGCGGGTTGGCGACCCTCTGTCCCGACCATTGTATGACGTGTGAAGCCCTACCCATAAGGGCCATGAGGACTTGACGTCATCCCCACCTTCCTCCGGTTTGTCACCGGCAGTCTCATTAGAGTGCTCTTGCGTAGCAACTAATGACAAGGGTTGCGCTCGTTGCGGGACTTAACCCAACATCTCACGACACGAGCTGACGACAGCCATGCAGCACCTGTGTTCCGGTTCTCTTGCGAGCACGGCCAAATCTCTTCGGCTTTCCAGACATGTCAAGGGTAGGTAAGGTTTTTCGCGTTGCATCGAATTAATCCACATCATCCACCGCTTGTGCGGGTCCCCGTCAATTCCTTTGAGTTTTAATCTTGCGACCGTACTCCCCAGGCGGTCCACTTCACGCGTTAGCTGCGCTACTAAGGCCTAACGGCCCCAACAGCTAGTTGACATCGTTTAGGGCGTGGACTACCAGGGTATCTAATCCTGTTTGCTCCCCACGCTTTCGTGCCTGAGCGTCAGTATTATCCCAGGGGGCTGCCTTCGCCATCGGTATTCCTCCACATATCTACGCATTTCACTGCTACACGTGGAATTCTACCCCCCTCTGACATACTCTAGCTCGGCAGTTAAAAATGCAGTTCCAAGGTTGAGCCCTGGGATTTCACATCTTTCTTTCCGAACCGCCTACACACGCTTTACGCCCAGTAATTCCGATTAACGCTTGC
*Bacillus siamensis* (Rt-11)	TCCTAGAGGTACCTCACCGACTTCGGGGGTTACAAACTCTCGGGGGGGGACGGGCGGGGTGTACAAGGCCCGGGAACGTATTCACCGCGGCATGCTGATCCGCGATTACTAGCGATTCCAGCTTCACGCAGTCGAGTTGCAAACTGCGATCCGAACTGAAAACAGATTTGTGGGATTGGCTTAACCTCGCGGTTTCGCTGCCCTTTGTTCTGTCCATTGTAGCACGTGTGTAGCCCAGGTCATAAGGGGCATGATGATTTGACGTCATCCCCACCTTCCTCCGGTTTGTCACCGGCAGTCACCTTAAAGTGCCCAACTGAATGCTGGCAACTAAGATCAAGGGTTGCGCTCGTTGCGGGACTTAACCCAACATCTCACGACACGAGCTGACGACAACCATGCACCACCTGTCACTCTGCCCCCGAAGGGGACGTCCTATCTCTAGGATTGTCAGAGGATGTCAAGACCTGGTAAGGTTCTTCGCGTTGCTTCGAATTAAACCACATGCTCCACCGCTTGTGCGGGCCCCCGTCAATTCCTCTTAGTTTCTGTCTTGCGACCGTACTCTCCAGAGCGGAGTGCTCAATGCGTTTTCTGCAGCACTAAGCCCGCGGAAACCCCCTAACACTTATCACTCATCGTTTACGACGTGGACTACCAGGGAATCTAAACTGTTCGGTCCCCGCCGGGTTTTTTTTTTTTTCATTTATTTTTGTCCAGAGAGTCCCCCTCCGCCACTGGTGTTTCCTTCCACATCTCTACGCATTTTCACCGCTACCCGTGGGATTTTCTTCTCTTCCTTCTTCTGCATCTCTATGTTCCCCAGTTTTTCCAATTGAACCTTCCCCCGGGGTTGAGCCGGGGGGGCTTTTCTCACATCAAAACTTAAAGAAAACCCGGCCCTGCCGAGACCCTTTTACCGCCCATTCTAATTTTCTCGGCGCT
