# Supplementary material for: Evaluation of the Efficacy of Three Antagonistic Bacteria Strains in the Management of Fire Blight
Source: Int J Mol Sci. 2025 May 7;26(9):4438. doi: 10.3390/ijms26094438 (PMC12072930; doi:10.3390/ijms26094438)
Supplement: Supplementary file 1 [file ijms-26-04438-s001.zip › ijms-3581146-supplementary.pdf]

Supplementary Table S1 Changes in MDA content in detached leaves after treatment with three types of antagonistic bacteria.

Experimental treatment	2d	4d	6d	8d
CK	5.18±0.55e	4.57±0.49f	3.66±0.33h	4.64±0.42de
EA	10.73±0.42a	13.34±0.72a	12.36±0.49a	9.98±0.36a
P-7	7.48±0.51cd	10.09±0.38c	7.91±0.38d	7.95±0.49b
P-10	4.05±0.43f	5.39±0.48f	4.77±0.42g	3.21±0.50f
P-11	6.57±0.50d	7.19±0.46e	6.98±0.43e	5.58±0.52d
T-7	9.52±0.43b	11.74±0.46b	9.97±0.47b	8.77±0.55b
T-10	8.26±0.54c	8.63±0.46d	8.90±0.45c	6.88±0.48c
T-11	5.62±0.48de	6.52±0.41e	5.74±0.46f	4.11±0.38e

Note: T-7, T-10, and T-11 respectively represent the therapeutic tests of *Leuconostoc mesenteroide*, *Alcaligenes faecalis*, and *Bacillus siamensis,* while P-7, P-10, and P-11 respectively represent the protective tests of *Leuconostoc mesenteroide*, *Alcaligenes faecalis*, and *Bacillus siamensis.* EA was used for the treatment of *Erwinia amylovora* only by inoculation, while CK represents the treatment of a single inoculation with clear water. Similar letter designations among means indicate no statistically significant differences (Tukey $P < 0.05$). The expression form of the data was: average value ± standard deviations. The same applies below.

Supplementary Table S2 Changes in PAL enzyme activity in detached leaves after treatment with three types of antagonistic bacteria.

Experimental treatment	2d	4d	6d	8d
CK	125.94±11.24f	111.11±9.16g	113.33±12.24e	117.00±11.98f
EA	145.33±10.99e	142.06±9.38f	158.50±12.34f	144.50±9.10e
P-7	271.06±13.38b	314.11±15.52b	278.17±11.50b	274.00±11.35b
P-10	301.83±14.57a	356.89±18.59a	336.33±18.58a	311.72±14.47a
P-11	239.11±13.21c	270.44±14.91c	249.67±12.08c	219.44±13.12c
T-7	215.67±12.89c	246.00±15.86c	227.94±11.58c	241.00±12.41c
T-10	164.72±11.78de	185.06±10.95e	172.39±10.15e	165.94±12.33de
T-11	184.22±10.96d	212.00±12.01d	197.17±11.19d	187.00±14.68d

Supplementary Table S3 Changes in CAT enzyme activity in detached leaves after treatment with three types of antagonistic bacteria.

Experimental treatment	2d	4d	6d	8d
CK	213.89±18.57f	234.07±20.56g	217.78±24.41g	201.67±21.03g
EA	265.19±16.91g	328.52±24.35f	285.37±21.17f	265.56±20.57f
P-7	454.81±17.50b	543.89±23.41b	455.74±21.76b	458.70±16.86b
P-10	530.37±26.11a	609.26±18.72a	509.63±19.95a	524.07±21.11a
P-11	411.67±17.41c	478.15±20.34c	421.30±15.16bc	417.59±16.88c
T-7	377.96±18.47cd	432.96±28.62cd	387.59±23.01cd	351.22±21.39d
T-10	303.52±18.16de	364.81±21.52ef	338.89±18.92e	382.22±22.46cd
T-11	342.22±25.92e	397.96±16.95de	362.41±22.26de	308.67±16.10e

Supplementary Table S4 Changes in POD enzyme activity in detached leaves after treatment with three types of antagonistic bacteria.

Experimental treatment	2d	4d	6d	8d
CK	1353.75±99.13f	1386.11±126.83g	1403.61±142.93g	1261.39±106.89f
EA	1575.28±72.00e	1863.06±114.75f	1983.06±90.25f	1580.42±73.33e
P-7	2571.39±82.04b	2829.86±92.84b	2983.33±101.52b	2204.44±87.63d
P-10	2869.86±94.33a	3073.61±114.86a	3352.78±79.53a	2986.67±90.04a
P-11	2396.81±86.99b	2482.08±79.30c	2697.50±95.70c	2469.72±88.07b
T-7	2201.81±96.64c	2436.67±104.00cd	2560.83±70.90cd	2462.92±93.38bc
T-10	1861.53±100.80d	2132.78±79.06e	2248.33±96.04e	2323.06±94.34cd
T-11	1980.14±91.59d	2317.78±84.97d	2440.42±74.66d	2342.22±77.75c

Supplementary Table S5 Changes in PPO enzyme activity in detached leaves after treatment with three types of antagonistic bacteria.

Experimental treatment	2d	4d	6d	8d
CK	121.77±5.54g	131.99±4.18f	127.97±4.46g	128.64±4.39g
EA	134.62±3.83f	121.97±4.37g	138.99±4.56f	141.90±5.47f
P-7	208.82±5.11b	174.63±4.02c	177.82±4.49b	187.42±5.35b
P-10	223.70±6.43a	199.40±5.51a	190.23±4.77a	201.63±6.67a
P-11	189.44±4.33c	186.03±4.84b	165.85±5.44c	176.13±4.64c
T-7	177.04±5.92d	141.32±4.16e	153.54±4.24de	164.94±5.84d
T-10	146.52±6.86e	152.55±4.57d	147.50±5.92ef	154.55±5.77e
T-11	158.61±6.29e	160.97±5.44d	161.02±3.36cd	159.31±6.05de

Supplementary Table S6 Changes in SOD enzyme activity in detached leaves after treatment with three types of antagonistic bacteria.

Experimental treatment	2d	4d	6d	8d
CK	612.56±35.32g	638.08±82.20f	656.46±47.08g	624.81±51.16g
EA	783.15±49.00e	892.58±56.68e	1028.56±61.36e	802.37±57.16f
P-7	1374.12±68.01a	1467.26±55.92b	1505.87±49.36b	1399.69±69.81b
P-10	1223.02±67.40b	1731.39±63.75a	1631.42±58.30a	1594.69±46.08a
P-11	1081.53±51.16c	1286.69±61.17c	1366.40±59.89c	1238.36±68.21c
T-7	981.11±78.35cd	1181.75±59.89c	1246.95±72.47cd	1146.50±64.71cd
T-10	728.02±46.25ef	974.59±46.25de	875.85±65.09f	949.91±62.02e
T-11	906.58±64.32d	1057.89±44.63d	1124.49±68.11de	1049.98±60.70de

Supplementary Table S7 Changes in MDA content in detached branches after treatment with three types of antagonistic bacteria.

Experimental treatment	2d	4d	6d	8d
CK	1.71±0.41g	3.24±0.44g	3.18±0.37f	3.38±0.43f
EA	6.45±0.42a	9.64±0.43b	11.23±0.57a	8.87±0.43a
P-7	3.94±0.41cd	6.26±0.38d	7.81±0.42bc	4.37±0.40e
P-10	3.27±0.35de	5.12±0.28e	4.76±0.41e	5.97±0.39cd
P-11	2.64±0.42ef	4.11±0.34f	4.25±0.45e	2.82±0.32f
T-7	7.28±0.49a	10.79±0.47a	8.50±0.39b	7.40±0.37b
T-10	5.52±0.34b	7.42±0.56c	6.95±0.55c	6.46±0.29c
T-11	4.69±0.40c	8.45±0.54c	5.81±0.39d	5.26±0.37d

Supplementary Table S8 Changes in PAL enzyme activity in detached branches after treatment with three types of antagonistic bacteria.

Experimental treatment	2d	4d	6d	8d
CK	103.61±6.46g	109.22±5.53g	115.11±7.61f	116.50±8.35g
EA	129.83±7.05f	130.56±5.49f	139.06±6.25e	142.78±8.18f
P-7	185.06±5.01b	206.00±6.82b	216.94±7.43b	211.44±7.01b
P-10	167.83±4.29cd	190.61±6.45c	194.51±5.41cd	178.61±5.20d
P-11	200.56±6.34a	222.78±7.81a	234.39±5.04a	240.39±8.65a
T-7	176.28±7.42bc	181.50±5.40cd	201.72±5.60c	194.17±6.76c
T-10	146.22±5.70e	143.78±6.50e	165.89±7.85d	156.00±6.48ef
T-11	159.17±5.66d	168.28±6.20d	177.44±6.91d	166.39±8.27de

Supplementary Table S9 Changes in CAT enzyme activity in detached branches after treatment with three types of antagonistic bacteria.

Experimental treatment	2d	4d	6d	8d
CK	21.30±1.76g	23.39±2.75g	21.80±1.89g	20.02±1.40g
EA	29.09±2.32f	32.48±1.49f	28.56±2.83f	26.33±1.97f
P-7	44.07±1.83b	53.87±2.41b	48.93±2.22b	45.67±1.86bc
P-10	40.87±1.85bc	47.30±1.86c	43.93±1.40c	41.98±2.32c
P-11	53.26±1.86a	61.02±2.14a	55.50±2.27a	52.48±2.01a
T-7	38.31±1.85cd	41.33±2.42d	39.46±2.07d	37.39±1.74d
T-10	33.35±2.22e	36.48±2.15e	33.89±1.89e	35.72±2.25de
T-11	34.78±1.49de	38.35±2.29de	35.69±2.11de	33.67±1.55e

Supplementary Table S10 Changes in POD enzyme activity in detached branches after treatment with three types of antagonistic bacteria.

Experimental treatment	2d	4d	6d	8d
CK	447.22±18.11f	467.78±32.10h	514.17±35.65e	551.57±31.65d
EA	619.91±38.17d	569.44±30.83g	539.81±32.42e	451.48±28.43ef
P-7	981.85±36.81b	1229.26±55.42b	973.15±51.16b	791.85±41.26b
P-10	885.00±35.32c	993.61±65.43c	816.39±38.74c	738.98±33.24b
P-11	1101.94±44.99a	1392.04±69.72a	1183.89±57.37a	916.20±47.35a
T-7	688.15±50.19d	848.43±44.45d	728.24±37.96d	639.09±34.96c
T-10	484.54±35.80ef	663.89±42.44f	418.70±45.23f	409.07±38.23f
T-11	523.24±41.99e	747.13±28.62e	621.85±39.47e	482.59±43.36de

Supplementary Table S11 Changes in PPO enzyme activity in detached branches after treatment with three types of antagonistic bacteria.

Experimental treatment	2d	4d	6d	8d
CK	133.68±5.05f	161.64±5.15f	130.85±6.27e	135.73±4.48f
EA	127.08±5.62f	145.71±7.10g	148.05±5.45d	124.19±4.98g
P-7	213.06±5.68b	237.28±7.36b	199.16±6.14b	207.98±6.32b
P-10	195.39±6.42c	221.57±7.30c	184.60±5.30c	177.81±7.35d
P-11	231.90±7.86a	266.20±8.22a	224.35±5.70a	225.76±6.72a
T-7	178.05±7.26d	205.66±7.28d	210.24±7.34b	191.60±4.80c
T-10	147.75±5.64e	175.11±6.03e	157.01±7.21d	147.43±6.22e
T-11	164.72±7.42d	187.84±8.90e	176.00±4.51c	161.06±8.77e

Supplementary Table S12 Changes in SOD enzyme activity in detached branches after treatment with three types of antagonistic bacteria.

Experimental treatment	2d	4d	6d	8d
CK	278.76±31.57g	353.29±49.29g	374.73±43.12f	363.50±38.30f
EA	544.99±39.82e	730.59±50.54e	680.96±39.86e	587.56±41.43d
P-7	866.93±45.15b	1119.88±44.42b	1208.73±38.38a	891.25±42.92b
P-10	766.69±41.43c	1019.65±45.94c	880.04±45.98d	741.19±33.66c
P-11	982.48±49.04a	1237.37±39.82a	1103.13±36.41b	993.68±44.76a
T-7	615.02±40.91de	839.23±39.97d	988.88±46.67c	827.97±34.77b
T-10	419.84±46.55f	596.06±44.49f	613.58±39.86e	496.41±35.50e
T-11	675.86±41.36d	913.73±43.16d	806.95±36.75d	650.96±35.76d
